# Accelerating skin wound healing by M-CSF through generating SSEA-1 and -3 stem cells in the injured sites

**DOI:** 10.1038/srep28979

**Published:** 2016-07-01

**Authors:** Yunyuan Li, Reza Baradar Jalili, Aziz Ghahary

**Affiliations:** 1 Department of Surgery, University of British Columbia, Vancouver, BC, V5Z 1M9 Canada

## Abstract

Wound healing is a complicated process requiring the collaborative efforts of different cell lineages. Our recent studies have found that one subset of hematopoietic cells can be induced to dedifferentiate into multipotent stem cells by means of a proliferating fibroblast releasable factor, M-CSF. Understanding the importance of stem cells on skin wound healing, here we evaluate the biological significance of M-CSF on skin wound healing. In an *in vivo* mouse skin excisional wound model, we found that SSEA-positive stem cells were present in wounded but not normal skin. After isolating skin cells from either normal or wounded skin by collagenase digestion, and analyzing the SSEA-1 positive cells by flow cytometry, we found a significant increase in the number of SSEA-1 positive cells in wounded skin. Topical application of M-CSF in skin wounds accelerated healing remarkably, while application of M-CSF-neutralizing antibody slowed wound healing. Furthermore, injection of EGFP-labeled hematopoietic cell-derived stem cells generated from M-CSF treated splenocytes resulted in EGFP-labeled cells being enriched in the skin wound site and further differentiated into functional organ-specific cells. Together, these data demonstrated that M-CSF makes a significant contribution to the healing process by inducing hematopoietic cell dedifferentiation into stem cells.

Skin wound healing proceeds through several overlapping patterns of events: coagulation, inflammation response, migration and proliferation of local resident cells, and tissue remodeling. The inflammation phase begins at the time of injury and lasts for 24 to 48 hours. In this phase, neutrophils and macrophages infiltrate from circulation into the wound site and cooperate to remove necrotic tissue, debris, and bacteria from the wound. CD4^+^ T lymphocytes including regulatory T cells also infiltrate to the wound site, but their role in wound healing is still unclear. In the migration and proliferation phase, epithelial cells and fibroblasts migrate from the edge of the wound toward the wound site and proliferate after receiving signals from platelets and inflammatory cells. The last phase of healing is tissue remodeling, beginning at about two to three weeks and lasting up to two years. Wound healing largely relies on the coordinated activation of resident cells and the infiltration of blood cells^1^. In addition, endogenous adult stem cells are considered to be key contributors to replenishing lost cells after injury. Studies have shown that adult stem cells could contribute to liver regeneration^2^,^3^, lung regeneration^4^,^5^, neuron regeneration^6^,^7^, heart repair^8^,^9^ and kidney repair^10^,^11^. Under the skin, after injury, stem cells from hair follicles^12^ and sweat glands^13^ at the edge of the uninjured area can migrate into the wound site and help support re-epithelialization and granulation.

Hematopoietic stem cells or hematopoietic cells have been suggested as having the capacity to trans-differentiate into organ-specific cells after tissue injury^14^,^15^,^16^,^17^,^18^ although this conclusion is still controversial^19^,^20^,^21^,^22^,^23^. We have recently identified a proliferating fibroblast-releasable factor, macrophage colony-stimulating factor (M-CSF), which can directly induce a subset of hematopoietic cells to be dedifferentiated into multipotent stem cells that are positive for stage-specific embryonic antigen-1 and -3 (SSEA-1 and SSEA-3) at the physiological concentration^24^. We have demonstrated that these hematopoietic cell-derived multipotent stem cells do in fact have the capacity to be differentiated into the cell type of three germ layers *in vitro*^24^.

SSEAs are carbohydrate epitopes associated with the lacto- and globo-series glycolipids SSEA-1, SSEA-3 and SSEA-4, and recognized respectively by three monoclonal antibodies^25^. SSEA-1 is expressed in murine embryos at the pre-implantation stage and teratocarcinoma stem cells, but is absent in human embryonic stem cells (ESC) and human embryonic carcinoma^25^,^26^. Both SSEA-3 and SSEA-4 are expressed in human ESC and teratocarcinoma stem cells, but SSEA-4 expression is absent in murine ESC^27^,^28^.

SSEAs have been used for cell markers of not only ESC but also induced pluripotent stem cells (iPSC) and multipotent stem cells^29^,^30^. A recent study showed that a rare subpopulation of SSEA-3-expressing cells exists in the dermis of adult human skin and these cell populations undergo a significant increase in cell number in response to injury^31^. However, the source and the functionality of this rare population of SSEA-3 cells are still unknown. Considering (i) the expression of SSEA-1 and-3 in our recently identified hematopoietic cell-derived multipotent stem cells, and (ii) that the factor to induce hematopoietic cell dedifferentiation is expressed and released from proliferating skin fibroblasts, in this study therefore we aimed to investigate whether SSEA-positive, hematopoietic cell-derived stem cells are present in the wound site and whether there is correlation among skin injury, M-CSF at the wound environment, SSEA-positive cells in the wound site, and skin wound healing. We also study the contribution of hematopoietic cell-derived stem cells to skin wound healing by injection of EGFP-labeled cells.

## Results

### SSEA-1 and -3 positive cells are present at the wound site after skin injury

**Figure 1:**
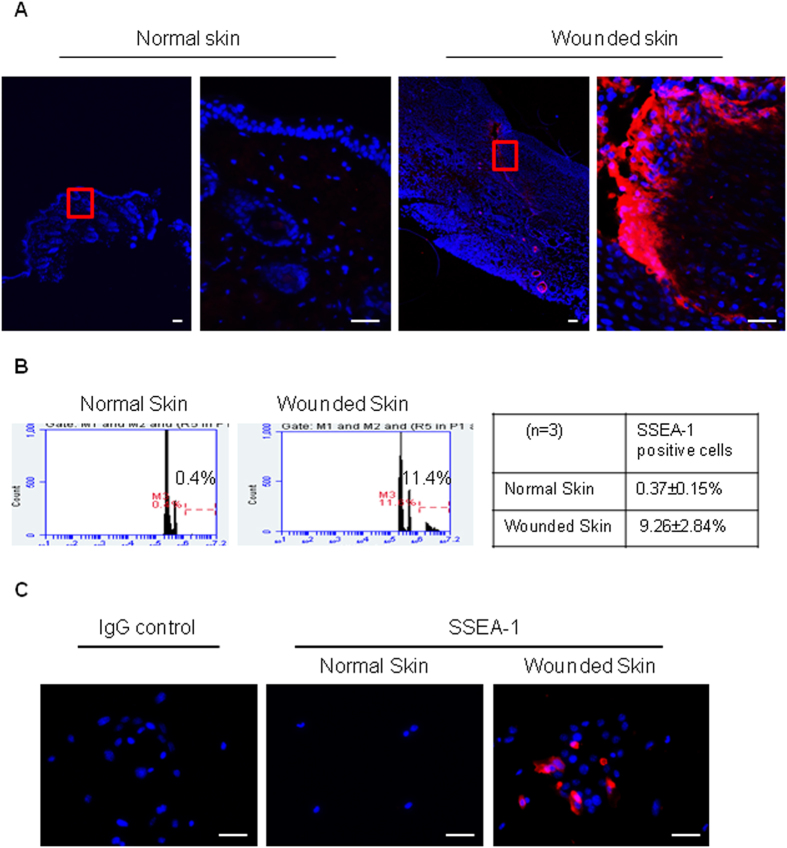
Presence of SSEA-1 positive stem cells in the injured skin. (**A**) Skin sections from surrounding normal or injured area (7 days post-operation) were stained with SSEA-1 antibody. SSEA-1 positive cells were indicated as red color and DAPI, a cellular nucleus marker was stained as blue. Scale bars, 50 μm. (**B**) Flow cytometry analysis of SSEA-1 positive cells which were isolated from either normal skin or injured skin (7 days post-operation) (*p* < 0.001, n = 3, comparison the percentage of SSEA-1 positive cell between normal skin and wounded skin). (**C**) 48-hours culture cells isolated from injured skin (7 days post-operation) were stained with SSEA-1 antibody. SSEA-1 positive cells are shown as red color and DAPI, a cellular nucleus marker was stained as blue. Scale bars, 50 μm.

Since fibroblasts become activated and proliferate^32^, and several types of hematopoietic cells infiltrate into the wound site^33^ during healing, we are interested here to examine whether SSEA-positive multipotent stem cells are present in the wound site after injury. Skin wounds were created by full-thickness punch biopsy in mice. SSEA-1, a marker for various types of endogenous multipotent stem cells^30^,^34^,^35^, ESC and iPSC^36^, was used to detect multipotent stem cells. Indeed, we were able to detect SSEA-1 positive cells in wounded skin but not normal skin (Fig. 1A). After isolating single cells from wounded or normal skin of mice by collagenase, staining with SSEA-1 antibody, and analyzing by flow cytometry, we detected a significantly higher number of SSEA-1 positive cells in isolated skin cells from wounded skin, as compared to those from normal skin (9.26 ± 2.84% *vs* 0.37 ± 0.15%, P < 0.01, Fig. 1B). When cells isolated from either wounded or normal skin were cultured in a medium containing M-CSF for 48 hrs, and then stained with SSEA-1 antibody, we confirmed SSEA-1 positive cells could indeed be isolated from wounded but not normal skin (Fig. 1C). Taken together, these data imply that SSEA-1 positive multipotent stem cells are present in the wound site after injury.

**Figure 2:**
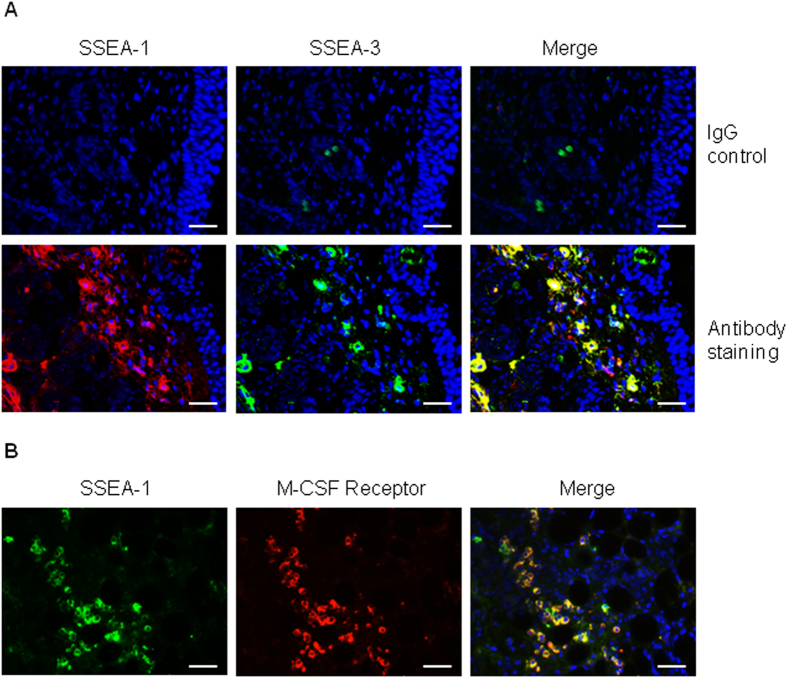
SSEA-1 positive cells are also SSEA-3 positive and express M-CSF receptor. (**A**) Injured skin sections were stained with SSEA-1 (red color) and SSEA-3 (green color) antibodies. IgG was used for a negative staining control. DAPI (blue) was used for cell nucleus staining. Scale bars, 50 μm. (**B**) Injured skin sections were stained with SSEA-1 (green color) and M-CSF receptor (red color) antibodies. DAPI (blue) was used for cell nucleus staining. Scale bars, 50 μm.

To further demonstrate that SSEA-1 positive cells in wound site are the same type of multipotent stem cells as described by our previous *in vitro* study^24^, we examined another marker, SSEA-3, which a number of studies have indicated as a marker for murine multipotent stem cells^36^,^37^,^38^. As expected, by using immunofluorescence staining we have here revealed that these SSEA-1 positive cells in wounded skin are also SSEA-3 positive (Fig. 2A). To further confirm SSEA-1 positive cells in wounded skin are the same type of stem cells induced by M-CSF as previously reported^24^, we examined the expression of the M-CSF receptor in SSEA-1 positive cells by staining wounded skin with both antibodies. As expected, we found the SSEA-1 positive cells are collocated with the M-CSF receptor in wounded skin (Fig. 2B), suggesting SSEA-positive stem cells in wounded skin are the same type of stem cells derived from hematopoietic cells, as previously reported.

To study whether SSEA-positive cells were proliferating, we stained wounded skin with SSEA-1 antibody and a proliferation marker, the antigen Ki67. Figure S1A showed many cells at the wound site are proliferating (Ki67 positive in nucleus staining). Similar to other cells in the wound site, the result showed that some of SSEA-1 positive stem cells were also Ki67-positive (Supplementary Figure S1B, SSEA-1 and Ki67 double positive cells).

**Figure 3:**
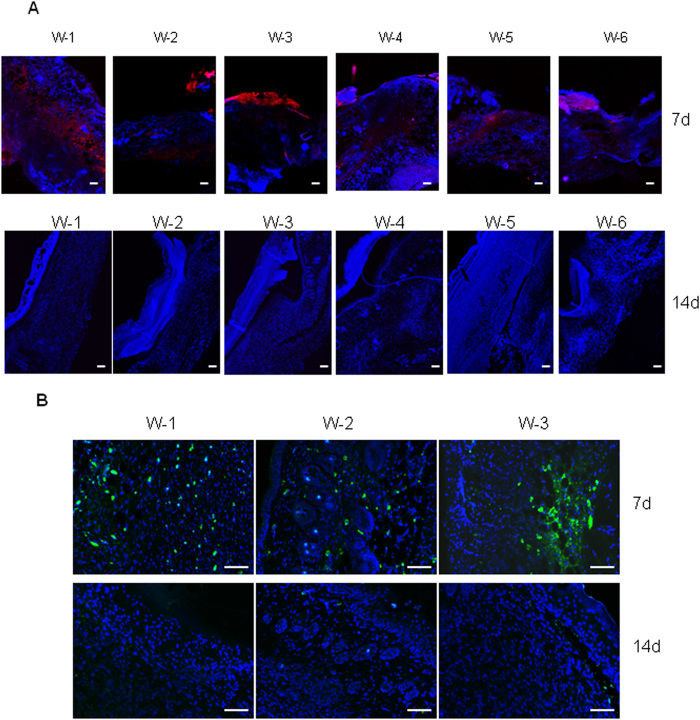
Injury-induced SSEA-1 or SSEA-3 positive cells are markedly reduced at the wounded area after wounds are fully healed. Wounds were created by punch biopsy (6 mm) in three mice. (**A**) After 7 days (top panels) and 14 days (bottom panels), respectively, six wounded skin samples (W 1 to W6) from three mice in each time point were fixed with 10% formalin, embedded in paraffin blocks and stained with SSEA-1 antibody (red). DAPI (blue) was used for cell nucleus staining. Scale bars, 50 μm. (**B**) Three wounds from 7 day and three wounds from 14 days injured skin were stained with SSEA-3 antibody. DAPI (blue) was used for cell nucleus staining. Scale bars, 50 μm.

We then compared the expression of SSEA-1 or SSEA-3 positive cells in wounds of seven days and fourteen days after injury. In our skin injury model, the wounds are usually healed around day ten after injury. Thus, wounds at day seven after injury represent unhealed wounds while wounds at day fourteen after injury represent healed wounds. As shown in Fig. 3A,B, when skin wounds are completely healed (as shown here on day fourteen) SSEA-1 or SSEA-3 positive cells are markedly reduced or absent at the wound site. To quantify the expression of SSEA-1, we measured the ratio of corrected total SSEA-1 fluorescence to corrected total DAPI fluorescence (SSEA-1/DAPI) in the wounds of day 7 and day 14, respectively, by using imageJ program. The result revealed a significant increase of SSEA-1 expression in wounds on day 7 as compared to that in wounds on day 14 [0.1784 ± 0.0643 (day 7) *vs* 0.0012 ± 0.0009 (day 14), ratio of CTCF of SSEA-1/CTCF of DAPI, n = 6, P < 0.001]. A possible explanation is that either SSEA-positive cells have differentiated into skin cells in which SSEA-1 and SSEA-3 are not expressed, or SSEA-positive cells have died as fibroblast-released M-CSF may reduce significantly after the wound is healed. In addition, our result revealed that the expression of M-CSF receptor in stem cells was found in wounds of seven days but not in wounds of fourteen days (Supplementary Figure S2), suggesting differentiated cells from SSEA-positive cells may not continue to need M-CSF to maintain their proliferation and survival.

### Topical application of M-CSF, or of M-CSF neutralizing antibody, changes the number of SSEA-1 positive cells in the wound site and the outcome of wound healing

**Figure 4:**
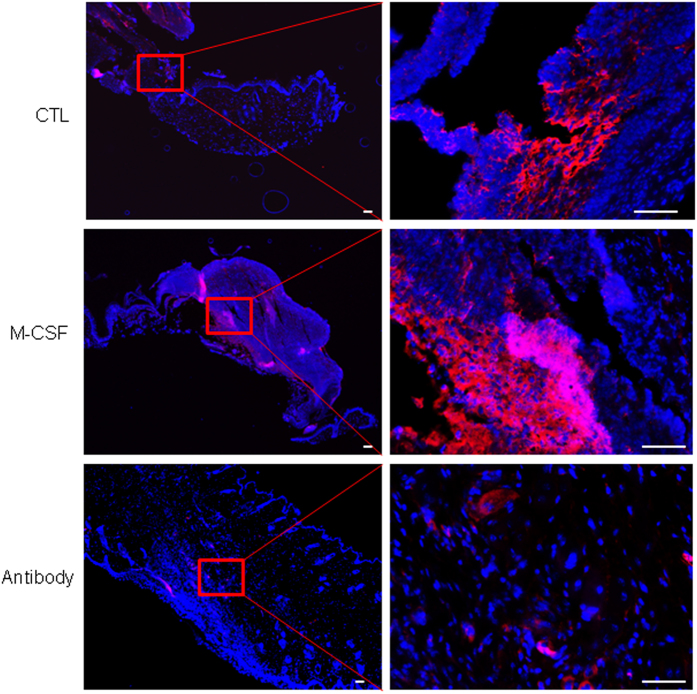
Injury-induced SSEA-1 positive cells were regulated by topical application of M-CSF or M-CSF neutralizing antibody. Skin wounds in mice were treated by either PBS control (top panels) or topical application of recombinant M-CSF protein (middle panels) or topical application of M-CSF neutralizing antibody for 6 days. This figure showed a representative skin section from 6 wounds in each group, stained with SSEA-1 (red color). DAPI (blue) was used for cell nucleus staining. Scale bars, 100 μm.

Next we investigated whether the presence of SSEA-1 positive stem cells is related to the presence of M-CSF at the wound environment. To do this, skin excisional wounds were topically administered with either a control of phosphate buffer saline (PBS), or M-CSF, or a neutralizing antibody against M-CSF, from day one until day six. The mice were sacrificed on day seven. Skin wounds were collected and fixed in 10% formalin for immunofluorescence staining with SSEA-1 antibody. As shown in Fig. 4, a higher number of SSEA-1 positive cells were seen in wounds receiving M-CSF treatment, compared to the number in untreated wounds. In concert with this result, the application of M-CSF neutralizing antibody remarkably reduced the number of SSEA-1 positive cells in the wound site, as compared to the number untreated wounds. These data suggest that the presence of SSEA-1 positive cells in the wound site can be affected by M-CSF at the wound environment, which normally is produced and released from proliferating fibroblasts, and some infiltrated immune cells, during the wound healing phase.

Since M-CSF can increase the number of SSEA-positive stem cells in the wound site, we were next interested to evaluate the effect of M-CSF on skin wound healing. As described above, skin excisional wounds in mice received either 0.1 ml PBS as controls, or 2 ng/0.1 ml of M-CSF, or 1 μg/0.1 ml of M-CSF neutralizing antibody per wound daily from day one to day nine. Wound size was measured on day one, day five and day ten, and the mice were sacrificed on day ten. Skin wounds were collected for histological evaluation.

**Figure 5:**
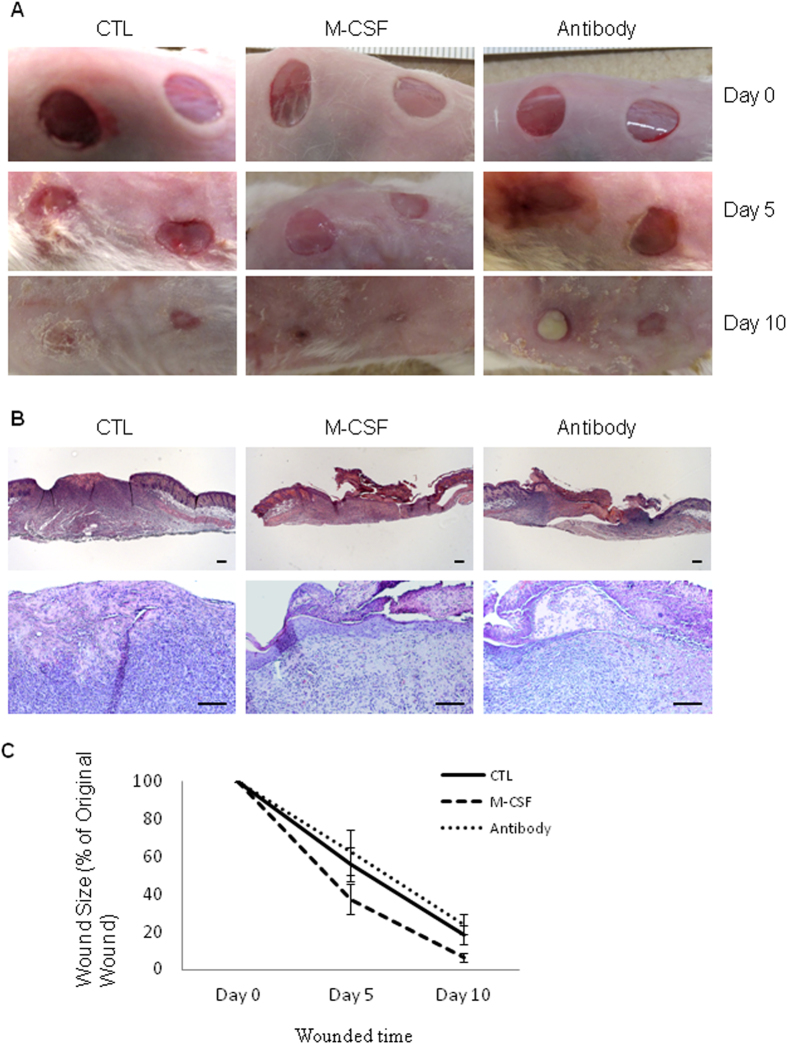
Skin wound healing was affected by topical application of M-CSF protein or M-CSF neutralizing antibody. Mouse skin wounds created by six millimetre punch biopsy, were treated either PBS (CTL) or M-CSF recombinant protein (M-CSF) or M-CSF neutralizing antibody (antibody) for *nine* days. Photos were taken and wound size was measured on day 0, day 5 and day10, respectively. Mice were sacrificed on day 10. Skin sections fixed by 10% formalin were stained with Hematoxylin and Eosin (H & E). (**A**) Skin wounds on different time point; (**B**) H & E staining. Scale bars, 100 μm; (**C**) Wound size in different time point.

As shown in Fig. 5, M-CSF treatments significantly accelerated skin wound healing as compared to untreated wounds on day five (55.9 ± 9.3% *vs* 37.5 ± 8.2% in wound size, control *vs* M-CSF, *p* < 0.0001, n = 12) and day ten (18.5 ± 4.8% *vs* 6.7 ± 2.3% in wound size, control *vs* M-CSF, *p* < 0.0001, n = 12). Consistent with this result, the topical application of a neutralizing antibody against M-CSF slowed skin wound healing as compared to controls on day five (55.9 ± 9.3% *vs* 62.4 ± 12.3% in wound size, control *vs* antibody of M-CSF, *p* = 0.102, n = 12) and day ten (18.5 ± 4.8% *vs* 24.0 ± 5.4% in wound size, control *vs* antibody of M-CSF, *p* = 0.0052, n = 12). These data demonstrated that M-CSF level in the wounds site can influence the outcome of skin wound healing.

### EGFP-labeled splenocyte-derived stem cells injected *via* tail vein are present in the wound site and can be differentiated into skin cells *in vivo*

**Figure 6:**
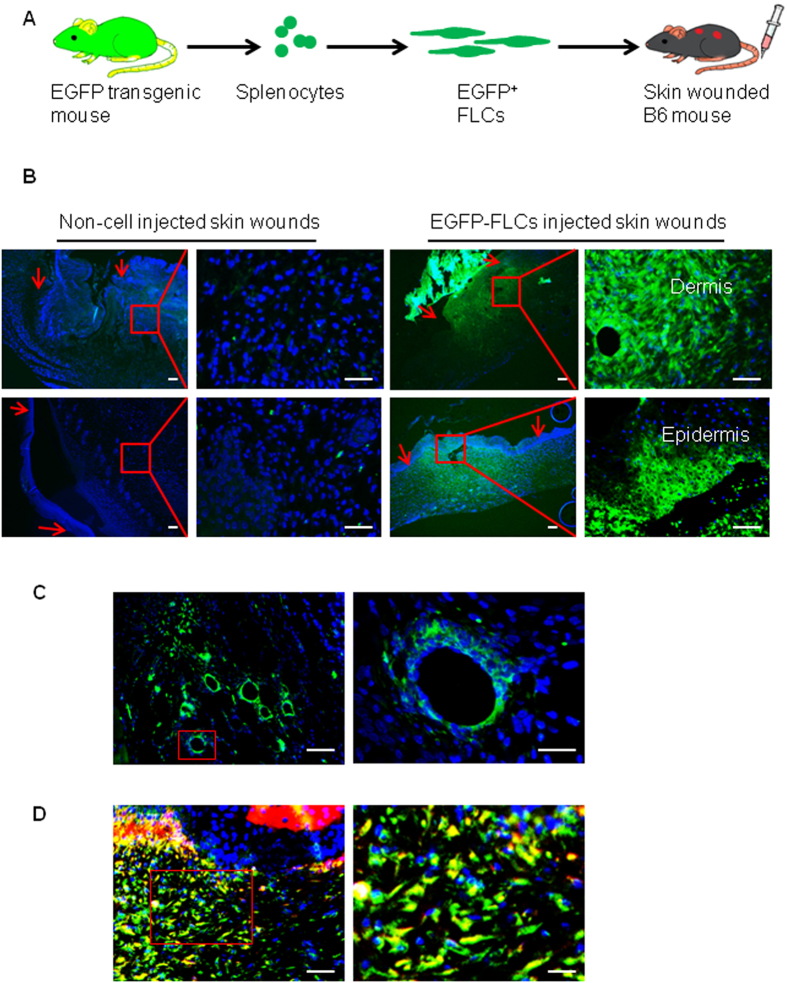
Presence of injected EGFP-labeled stem cells in the injury site and differentiation of EGFP-labeled stem cells into functional skin cells. (**A**) Schematic of the protocol for generation of EGFP^+^ stem cells (fibroblast like cells, FLCs), injection of EGFP^+^ stem cells into syngeneic mice. Splenocytes were obtained from EGFP transgenic mice and cultured in media containing M-CSF for 2 weeks to make hematopoietic cell-derived stem cells. Stem cells were then injected into the tail vein of the wounded mice. (**B**) EGFP expressing cells were detected in the injury sites but no surrounding normal skin of mice receiving EGFP-labeled splenocyte derived stem cells. The right top panel showed green cells in dermis while the right bottom panel shows green cells in epidermis of skin. DAPI (blue) was used for nucleus staining. (**C**) EGFP labeled stem cells were seen in blood vessel like structures from injured skin, DAPI (blue) (blue) was used for nucleus staining. Scale bars, 50 μm. (**D**) Skin sections stained with type 1 pro-collagen antibody (red color) demonstrated that EGFP expressing green cells in dermis express type 1 collagen. DAPI was used for cell nucleus staining. Scale bars, 50 μm.

Finally, to assess the differentiation potential of hematopoietic cell-derived stem cells (fibroblast-like cells, FLCs) *in vivo* and their contributions to wound healing, we cultured enhanced green fluorescence protein (EGFP) positive FLCs obtained from the splenocytes of UBC-EGFP transgenic mice by culture in a medium containing M-CSF. Three million EGFP^+^ FLCs per mouse were injected into the blood circulation of syngeneic mice *via* tail vein (Fig. 6A). Skin wounds were created by punch biopsy simultaneously. After fourteen days, blood, spleen, liver, heart, lung, and bone marrow cells, and both wound-surrounding normal skin and wounded skin were collected. Fluorescence microscopy failed to detect any EGFP-positive cells in spleen, lung, liver, heart or kidney of mice receiving EGFP stem cells (Supplementary Figure S3A), and flow cytometry similarly detected none in splenocytes or peripheral blood mononuclear cells (Supplementary Figure S3B). Furthermore, there were no EGFP-positive cells detected in the surrounding normal skin (Supplementary Figure S3C). However, EGFP-positive cells were found in the dermis and some parts of the epidermis of healed skin (Fig. 6B). Furthermore, our results revealed that injected splenocyte-derived multipotent stem cells could also differentiate to blood vessel-like structures in the wound sites (Fig. 6C). To examine whether EGFP-positive cells in the dermis could contribute to the skin healing process through differentiation into fibroblasts, we performed an immunofluorescence staining with type-1 pro-collagen antibody. While undifferentiated FLCs are known to be negative for type 1 pro-collagen^24^, results from this study revealed that almost all infiltrated EGFP-positive FLCs in dermis express type-1 pro-collagen (Fig. 6D), suggesting hematopoietic cell-derived stem cells are able to differentiate into functional skin cells.

## Discussion

Following our previous findings that hematopoietic cells can be dedifferentiated into SSEA-positive multipotent stem cells *in vitro*, induced by a proliferating fibroblast releasable factor M-CSF^24^, in this study we performed an *in vivo* investigation to examine whether tissue injury can induce hematopoietic cell dedifferentiation to SSEA-positive stem cells and whether these SSEA-positive stem cells participate in the process of healing skin wounds. By staining with SSEA-1 and-3 antibodies in injured skin, we demonstrated the presence of SSEA-1 and-3 positive stem cells in the skin at the injured site. Obviously, the number of SSEA-positive stem cells was regulated by the presence of M-CSF in the injury site. Our study further revealed that topical application of M-CSF protein on skin wounds accelerates healing while obstruction of M-CSF by a neutralizing antibody slows wound healing. We also demonstrated that an injection of hematopoietic cell-derived stem cells can be differentiated into organ-specific cells *in vivo*. Data obtained in a skin wound healing model thus point to a novel role for infiltrated hematopoietic cells in tissue repair.

M-CSF is a multifunctional pro-inflammatory cytokine involved in differentiation, proliferation and survival of monocyte progenitor cells^39^. Natural mutations of the M-CSF locus in mouse (*op*/*op*) and rat (*tl*/*tl*) result in reduction of macrophages and osteoclast density in tissues, toothlessness, severe growth retardation and low fertility^40^,^41^. These deficiencies can be partially restored by injection of recombinant human M-CSF^42^. A number of studies have shown that expression of M-CSF in local resident cells is markedly increased in response to tissue injury^43^,^44^,^45^,^46^. The up-regulation of M-CSF expression and release are correlated with the activation and proliferation of local resident cells. Our previous study demonstrated that the level of M-CSF in conditioned medium is significant higher when fibroblasts are in proliferation status, as compared to cells in quiescent status^24^. The increase of M-CSF expression in wound sites is beneficial to wound healing. We found that removing M-CSF at the skin wound environment, by topical application of M-CSF antibody, significantly slows wound healing while topical application of M-CSF recombinant protein accelerates skin wound healing. This result was strongly supported by other studies showing the effects of M-CSF on wound healing. Kawahara *et al* found that the healing of gastric ulcers was significantly delayed and the degree of vascularisation in the ulcerated area was significantly lower in M-CSF knockout *op*/*op* mice compared to normal mice^47^. Another study showed that application of M-CSF recombinant protein increased new granulation tissue formation in an ischemic dermal ulcer rabbit ear model^48^. A similar result was reported in an acute kidney injury mouse model^43^.

The mechanisms of M-CSF regulating wound healing are still unclear. We found that there was no notice effect of M-CSF protein on the expression of several extracellular matrix proteins [collagen-1 α1, collagen-3 α1 and α-smooth muscle actin (α-SMA)] and matrix metalloproteinases (MMP-13 and MMP-7) (Supplementary Figure S4). Previous studies have attributed the beneficial effect of M-CSF on wound healing to the increases of local macrophages and dendritic cells^43^,^47^,^48^. For example, one study found that genetic deletion of M-CSF by a pharmacologic inhibitor of C-fms, the receptor of M-CSF, resulted in both a marked reduction in the number of macrophages and dendritic cells and the slowing of recovery in an acute kidney injury model^43^. Another study showed that in op/op M-CSF deficient mice, the healing of gastric ulcers was significantly slower, and fewer macrophages was observed in the injured site, as compared to normal mice^47^. Although the data obtained from these studies suggested macrophages and dendritic cells might play a role in tissue repair through secreting cytokine and growth factors^43^, the evidence for correlation among macrophages, dendritic cells and wound healing is still lacking. As M-CSF is well known as a factor for proliferation, differentiation and survival of monocyte lineage cells, the higher number of macrophages and dendritic cells in injured tissues may be simply a side effect of M-CSF.

Based on our previous finding that M-CSF can induce hematopoietic cells to be dedifferentiated into SSEA-1 and-3 multipotent stem cells^24^, in this study we investigated whether SSEA-positive stem cells are present in injury site, and whether there is a correlation between M-CSF and SSEA-positive stem cells in injured tissue. In a skin wound healing mouse model we found skin injury can induce SSEA-1 positive stem cells from 0.37 ± 0.15% in normal skin to 9.26 ± 2.84% in injured skin. SSEA-1 positive cells are also SSEA-3 positive and express M-CSF receptor, suggesting these cells at the wound site are the same type of cells previously reported as hematopoietic cell-derived multipotent stem cells^24^. These cells may be also the same as previously reported SSEA-3 positive cells in the dermis^31^. The increase of SSEA-positive cell numbers at the wound site can be explained by the regulation of M-CSF to hematopoietic cell-derived stem cells: it is well known that tissue injury can activate quiescent fibroblasts to proliferate, and M-CSF is expressed and released from proliferating fibroblasts^24^. M-CSF at the wound site may directly induce one type of immune cell dedifferentiation into SSEA-1 and SSEA-3 positive stem cells. Indeed, applying a M-CSF neutralizing antibody to the wounds can markedly reduce the number of SSEA-1 positive cells and applying M-CSF recombinant protein can increase the number of SSEA-1 positive cells.

SSEA-positive stem cells contribute to skin wound healing: increasing the number of SSEA-positive cells by application of M-CSF recombinant protein enhances wound healing while application of a M-CSF neutralizing antibody slows healing. The contributions of SSEA-positive cells or M-CSF protein on skin wound healing can be explained by different mechanisms. SSEA-positive cells are hematopoietic cell-derived stem cells. As shown in Supplementary Figure S5C, undifferentiated cells can express interleukine-1β (IL-1β), IL-4, IL-10, transforming growth factor-β (TGF-β), tumor necrosis factor-α (TNF-α) and migrating inhibitory factor (MIF). Factors such as IL4, TGF-β and TNF-α have been suggested as accelerating wound healing^49^,^50^,^51^. We also found that differentiated and undifferentiated hematopoietic cell-derived multipotent stem cells express α-smooth muscle actin (Supplementary Figure S5B) which can increase wound contraction^52^. As hematopoietic cell-derived stem cells have multipotent differentiation capacity, they can differentiate into mesenchymal cells, epithelial cells and blood vesicle endothelial cells^24^ (Fig. 6 and Supplementary Figure S5A), which would provide a cell source and extracellular matrix for wound healing.

By injection of splenocyte-derived stem cells into circulation, we observed that EGFP-labeled splenocyte-derived multipotent stem cells were accumulated at the wound sites but not found in surrounding normal skin and other organs (Supplementary Figure S3). This enrichment may be due to a number of reasons: first of all, the survival and proliferation of hematopoietic cell-derived stem cells are absolutely M-CSF dependent^24^. In normal conditions, the concentration of M-CSF in skin and other organs is not enough to maintain the survival of this type of stem cell. However, when injury activates quiescent fibroblasts to express and release M-CSF, a high concentration of M-CSF will result at the wound environment, in which mobilized hematopoietic cell-derived stem cells can survive and further proliferate. Second, the enrichment of EGFP-labeled stem cells at the wound site might also be driven by one or more members of the constellation of chemokine receptors. A number of previous studies have confirmed different type of stem cells are recruited and enriched at damaged tissues^2^,^3^,^4^,^5^,^6^,^7^,^8^,^9^,^10^,^11^. Grafting hematopoietic stem cells into bone marrow or circulation has been found beneficial for injured tissues as these cells are trans-differentiated into organ-specialized cells^14^,^15^,^16^,^17^,^18^. Further study is needed to clarify what mechanism drives hematopoietic cell-derived stem cells to accumulate in injured tissues but not in normal organs and whether injury in other organs can also recruit such stem cells into the injury sites.

In conclusion, we have demonstrated a correlation among tissue injury, M-CSF, hematopoietic cell-derived multipotent stem cells and healing *in vivo.* Hematopoietic cell-derived SSEA-positive multipotent stem cells are an important but previously unrecognized mechanism in M-CSF regulating wound healing. This *in vivo* study further supported our previous *in vitro* finding that hematopoietic cells can be induced by M-CSF to dedifferentiate into multipotent stem cells.

## Methods

### Mouse skin wound healing assay

The animal experimental protocol was approved by the University of British Columbia Animal Care Committee. The methods were carried out in accordance with the approved guidelines. Mice used in this study were females eight to ten weeks old. Full-thickness skin excision wounds (four six-millimetre circular wounds per mouse) were created on the dorsal surface of C57BL/6 mice (Jackson Laboratory, ME, USA). Wounded skin and surrounding normal skin were collected after mice were sacrificed at the indicated time point. For observation of the effect of M-CSF or M-CSF antibody on SSEA-1 positive cells in the wound site, wounds were covered by Tegaterm dressing from day one until the end of the experiment and treated with either PBS (control) or 0.1 ml of M-CSF (14–8983–80, eBioscience) at a concentration of 20 ng/ml or 0.1 ml of M-CSF neutralizing antibody (AB-416-NA, R & D Biosystems) at a concentration of 10 μg/ml daily per wound from day one to day six. Solution was directly injected onto the top of wound and covered by Tegaderm. As the wound was open, we expected the solution would be taken up by the wound. After sacrifice, skin tissues including wounded and surrounding normal tissues were excised by punch biopsy. Skin samples were fixed with 10% formalin overnight and paraffin blocks were used for histological evaluation and immunofluorescence staining.

To investigate the effect of M-CSF on skin wound healing, nine mice were used to make skin wounds (four wounds per mouse) by a six-millimetre punch biopsy at the dorsal site. Wounds were covered by Tegaderm dressing and treated by either PBS (control) or 0.1 ml of M-CSF at a concentration of 20 ng/ml (eBioscience, Mouse M-CSF recombinant protein, 14–8983–80) or 0.1 ml of M-CSF neutralizing antibody at a concentration of 10 μg/ml (R & D Biosystems, Mouse M-CSF neutralization antibody, MAB4161) daily per wound as described above, from day one to day nine. Dressings were changed on day five. Wound sizes were measured on day zero, day five and day ten by marking the wound size with a transparent film and calculating the percentage of original size through an imaging program. Mice were sacrificed on day ten and skin samples were collected for histological analysis.

### Isolation and culture of cells from mouse wounded skin or normal skin

Four punch biopsies (6 mm) from wounded skin and eight punch biopsies from normal skin for each sacrificed mouse were taken. Tissue was minced into small pieces and incubated with type-1 collagenase (1 mg/ml in PBS, sigma) for 90 minutes in a shaker at 225 rpm and 37 °C. Tissue debris was removed by being passed through a 40 μm nylon filter. Cells were harvested by centrifugation at 500 × g for 5 minutes. Cells were either used for flow cytometry analysis or cultured in a mixture containing 50% fresh medium (DMEM containing 10% FBS) and 50% fibroblast conditioned medium (FCM) for 48 hrs before being fixed with 10% formalin and subjected to fluorescence staining with SSEA-1 antibody.

### Immunofluorescence staining

In this study, the following primary and secondary antibodies were used: Type-1 procollagen monoclonal antibody (SP1D8, 1:10, Developmental Studies Hybridoma Bank); SSEA-1 (MC480) antibody (4744, 1:200, Cell Signalling Technology); SSEA-3 (MC-631, IgM) antibody (MAB1434, 1:1000, R & D Systems); M-CSF receptor (CD115) antibody (14–1152, 1:50, eBioscience); Ki67 antibody (14–5698, 1:2000, eBioscience); Alexa Fluo568 goat anti-mouse IgG (A1104, 1:1000, Life Technology); Alexa Fluo488 goat anti-mouse IgG (A11029, 1:1000, Life Technology); Alexa Fluo488 goat anti-rat IgM (μ chain) (A21212, 1:1000, Life Technology); Rhodamine Red^TM^ X-conjugated affiniPure goat anti-rat IgG, 1:500, Jackson Immuno Research). Cells and skin sample were fixed by 10% formalin. For skin samples, 5 μm sections were used for staining. Some experiments needing to penetrate the cell membrane to detect intracellular protein used 0.2% Triton X-100 or 0.5% saponin. Cells or skin sections were then incubated with primary antibodies overnight after blocking with blocking solution containing 5% goat serum and 5% bovine serum albumin for one hour. After being washed three times with PBS or PBS-0.05% Tween-20 (PBS-T) at room temperature, cells were incubated with secondary fluorescently conjugated antibodies for another hour. After being washed three further times, cells were stained with 1.5% Vectashield Mounting medium with DAPI (Vector Laboratories) while slides were directly mounted with Vectashield Mounting Medium with DAPI. Images were captured using a Zeiss Axioplan 2 fluorescence microscope and AxioVision image analysis software. To calculate the corrected total fluorescence (CTCF) of SSEA-1 staining, we measured the area, cell fluorescence integrated density and mean gray value in selected field of wounds using imageJ program. The CTCF were calculated by the formula [CTCF = integrated density-(Area of selected cell X Mean fluorescence of background readings)].

### Flow cytometry

After dissociation, cells were washed twice with PBS and fixed with 10% formalin for thirty minutes at room temperature. After being washed three times with PBS, non-specific bindings were blocked by incubation with PBS containing 5% goat serum and 5% BSA for one hour at room temperature. Cells were then incubated with SSEA-1 primary antibody at a concentration of 1:500 for 45 minutes at room temperature. After being washed three times with PBS, cells were incubated with a fluorescence-conjugated secondary antibody for 45 minutes. After washing, cells were finally analyzed by flow cytometry (BD Acuuri C6).

### Culture enhanced green fluorescent protein (EGFP)-labeled hematopoietic cell-derived stem cells

Splenocytes from B6. UBC-EGFP transgenic mice (Jackson Laboratory, ME) were cultured in a medium containing 10 ng/ml of M-CSF for ten days. Adherent stem cells were used to inject into the mice as described below.

### Injection of FLCs into mice after skin injury

Full-thickness skin excision wounds (four six-millimetre circular wounds per mouse) were created on the dorsal surface of eight-week old female C57BL/6 mice (Jackson Laboratory). At the same time, three million EGFP-labeled splenocyte-derived stem cells were injected into the circulation through tail vein. On day fourteen, the mice were sacrificed, and skin tissues including wounded and surrounding normal tissues (6 mm) were excised. Tissues were fixed in 10% formalin and embedded in paraffin for immunofluorescence staining.

## Statistics

All data are presented as a mean ± standard deviation (SD). Data were analyzed using the analysis of variance Tukey-Kramer multi-comparisons test to compare the means between study groups and their controls. A *p* value of <0.05 was considered as a statistically significant difference between mean values.

## Additional Information

**How to cite this article**: Li, Y. *et al*. Accelerating skin wound healing by M-CSF through generating SSEA-1 and -3 stem cells in the injured sites. *Sci. Rep.* **6**, 28979; doi: 10.1038/srep28979 (2016).

## Supplementary Material

Supplementary Information
