# Supplementary material for: Accelerating skin wound healing by M-CSF through generating SSEA-1 and -3 stem cells in the injured sites
Source: Sci Rep. 2016 Jul 1;6:28979. doi: 10.1038/srep28979 (PMC4929493; doi:10.1038/srep28979)
Supplement: Supplementary Information [file srep28979-s1.pdf]

Accelerating skin wound healing by M-CSF through generating SSEA-1 and -3 stem cells in the injured sites

Yunyuan Li, Reza Baradar Jalili, Aziz Ghahary

Department of Surgery, University of British Columbia, Vancouver, BC, Canada, V5Z 1M9

Running Title: M-CSF accelerates skin wound healing

***Correspondence and requests of materials should be addressed to**:

Aziz Ghahary, PhD, Professor

4520 ICORD, 818 10th Avenue,

Vancouver, BC, CANADA V5Z 1M9

Tel: (604) 675-8862; Fax: (604) 675-8865

Email: aghahary@mail.ubc.ca

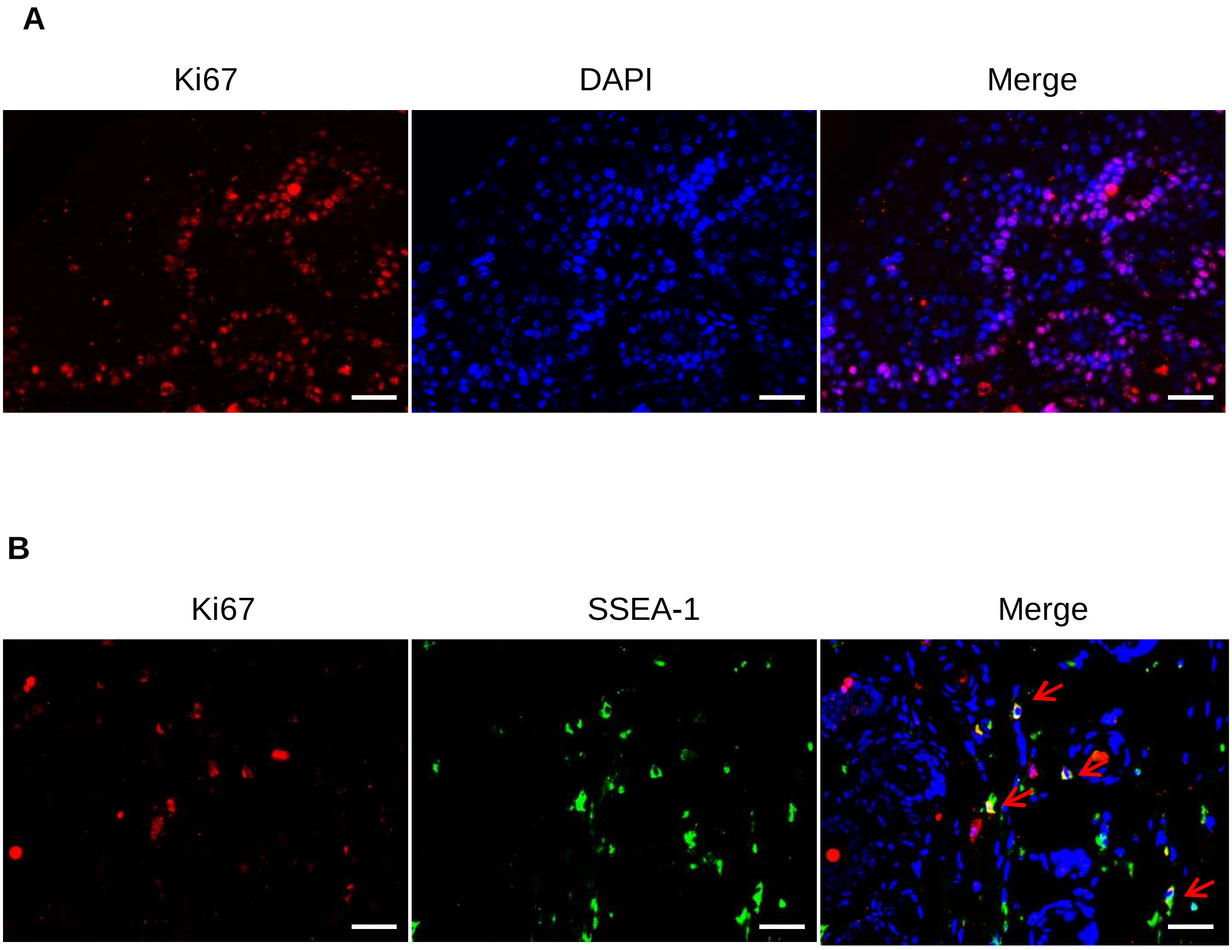

Figure S1. Detection of Ki67 proliferating cells in skin wounds. Sections from injured skin wounds on day 7 were fixed by 10% formalin and used for immunostaining. Sections were incubated with SSEA-1 (green color) and Ki67 (red color) antibodies. DAPI was used a cellular nucleus staining (blue color). Scar bars, 100 μm. (**A**) Ki67 positive cells in a healing wound. (**B**) Some of SSEA-1 positive cells are Ki67 positive (arrows indicated double positive cells).

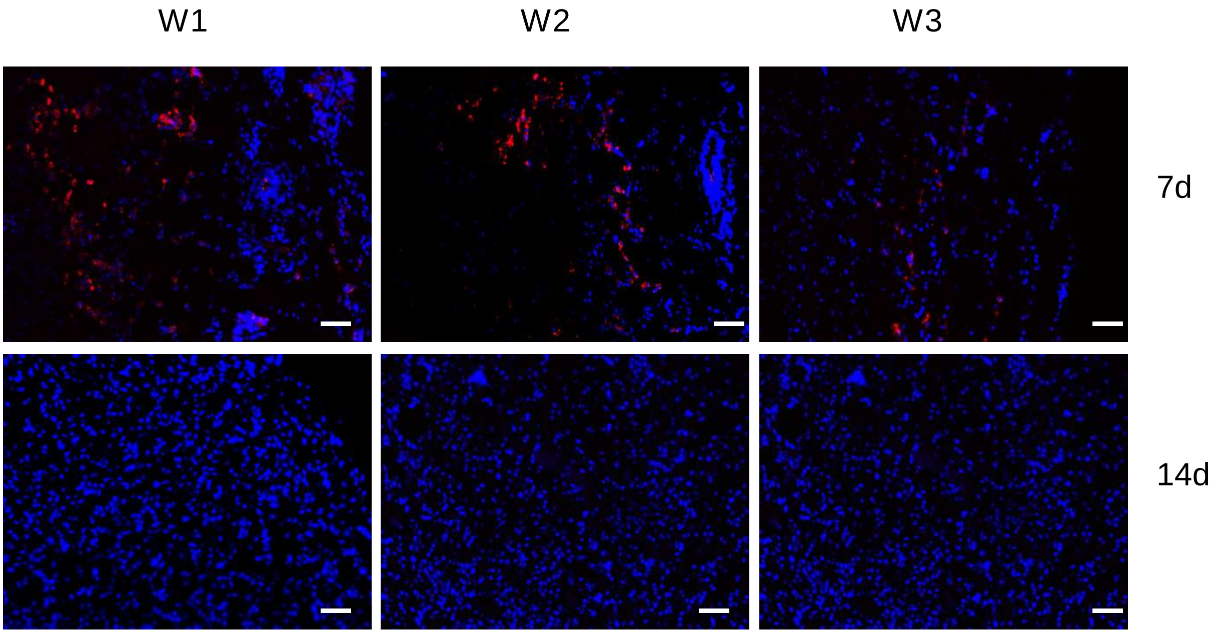

Figure S2: the expression of M-CSF receptor positive cells in skin wounds are reduce after wound is healed. Three sections from wounds on day 7 and day 14 were stained with SSEA-1 antibody (red color). DAPI was used a cellular nucleus staining. Scale bars, 100 μm.

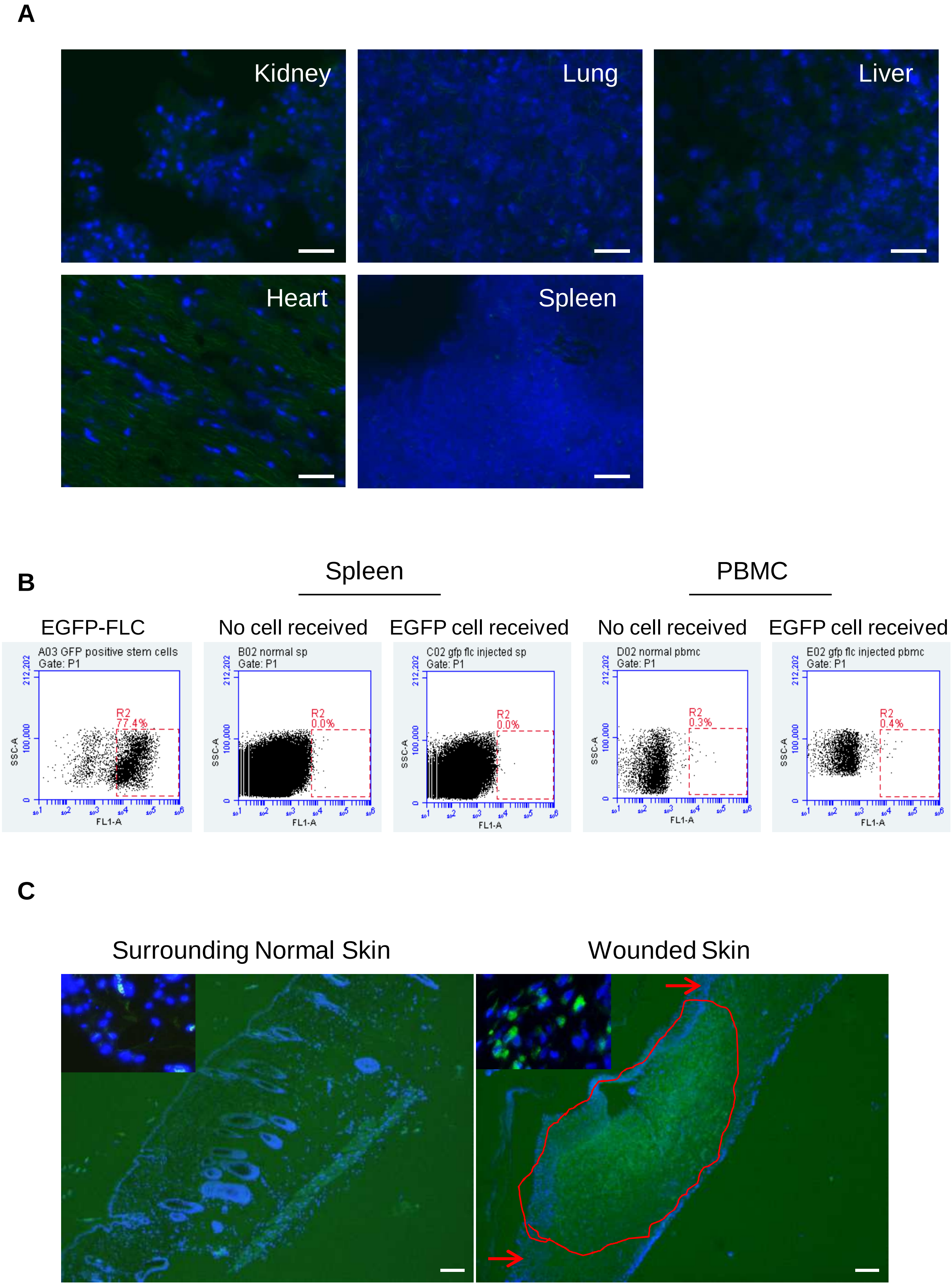
A
Kidney
Lung
Liver
Heart
Spleen
B
Spleen
PBMC
EGFP-FLC
No cell received
EGFP cell received
No cell received
EGFP cell received
A03 GFP positive stem cells
Gate: P1
R2
77.4%
B02 normal sp
Gate: P1
R2
0.0%
C02 gfp flc injected sp
Gate: P1
R2
0.0%
D02 normal pbmc
Gate: P1
R2
0.3%
E02 gfp flc injected pbmc
Gate: P1
R2
0.4%
SSC-A
FL1-A
C
Surrounding Normal Skin
Wounded Skin

Figure S3

Figure S3: EGFP-labeled stem cells are only seen in wound site of skin after injection into the circulation. Three million of EGFP –labeled splenocyte-derived multipotent stem cells were injected into the circulation of mouse. Skin wounds were created simultaneously. After 14 days, blood, bone marrow, spleen, lung, heart, liver, wound surrounding normal skin and wounded skin were collected to detect EGFP-labeled green cells. (**A**) Images of green fluorescence in cells from different organs. Scale Bars, 100 µm. all organs showed negative detection of EGFP-positive cells. (**B**) Flow cytometry analysis in peripheral blood mononuclear cells and bone marrow cells to detect EGFP-labeled cells. Results showed there was no EGFP –positive cells in spleen and PBMC of recipients. (**C**) Images of green fluorescence in wound surrounding normal skin and wounded skin. Scale bars, 100 µm. In wounded skin, wound was marked by red arrows and the EGFP-labeled stem cells recruitment area was marked by a red line. Results suggested injected EGFP-labeled M-CSF-treated splenocytes were enriched in wounded skins but not normal skins.

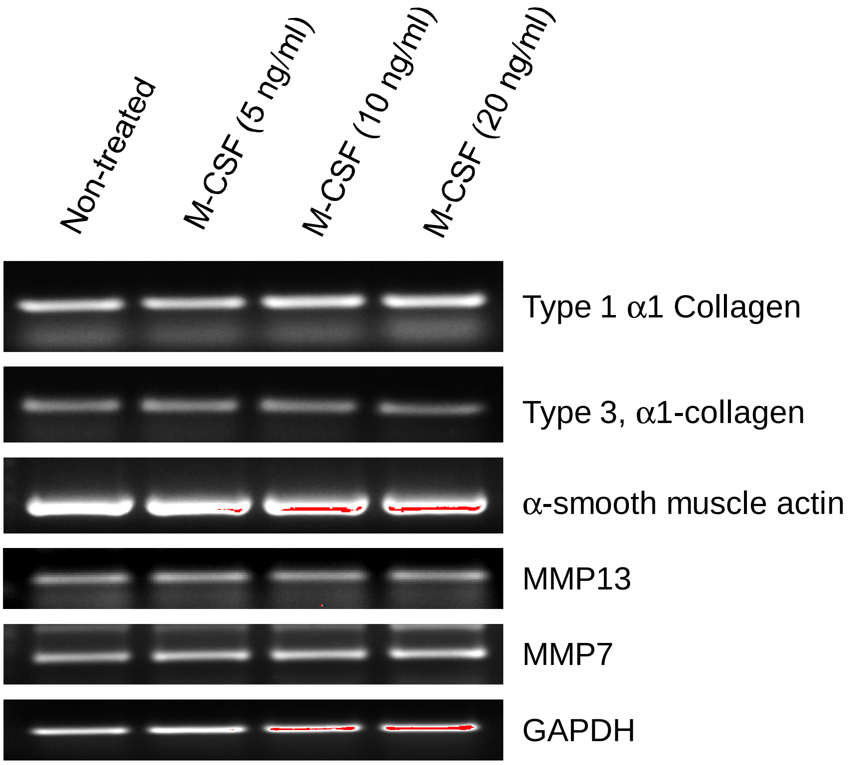

Figure S4. Effects of M-CSF on the expression of extracellular matrix and meta in mouse fibroblasts. Mouse fibroblasts at passage 3 were treated with 0, 5 ng/ml, 10 ng/ml or 20 ng/ml of recombinant mouse M-CSF protein for 48 hrs. Cells were harvested and cDNA was synthesized by Superscript III (Invitrogen). PCR were performed for 32 cycles. Primers for PCR were: collagen-1α1 (sense: gacgccatcaaggtctactg, antisense: acggaatccatcggtcg); collagen-3 α1 (sense: gaaaccccagcaaaacaaaa, antisense: tgacctgtattgggtggttg); α-SMA (sense: cgatagaacacggcatcata, antisense: catcaggcagttcgtagcc); MMP-7 (sense: acctaggtgtggagtgccag, antisense: tcccccaactaaccctcttg); MMP-13 (sense: ggtccaggcgatgaagacccca, antisense: gggtgcaggcgccagaagaa); GAPDH (ggcattgctctcaatgacaa, antisense: tgtgagggagatgctcagtg).

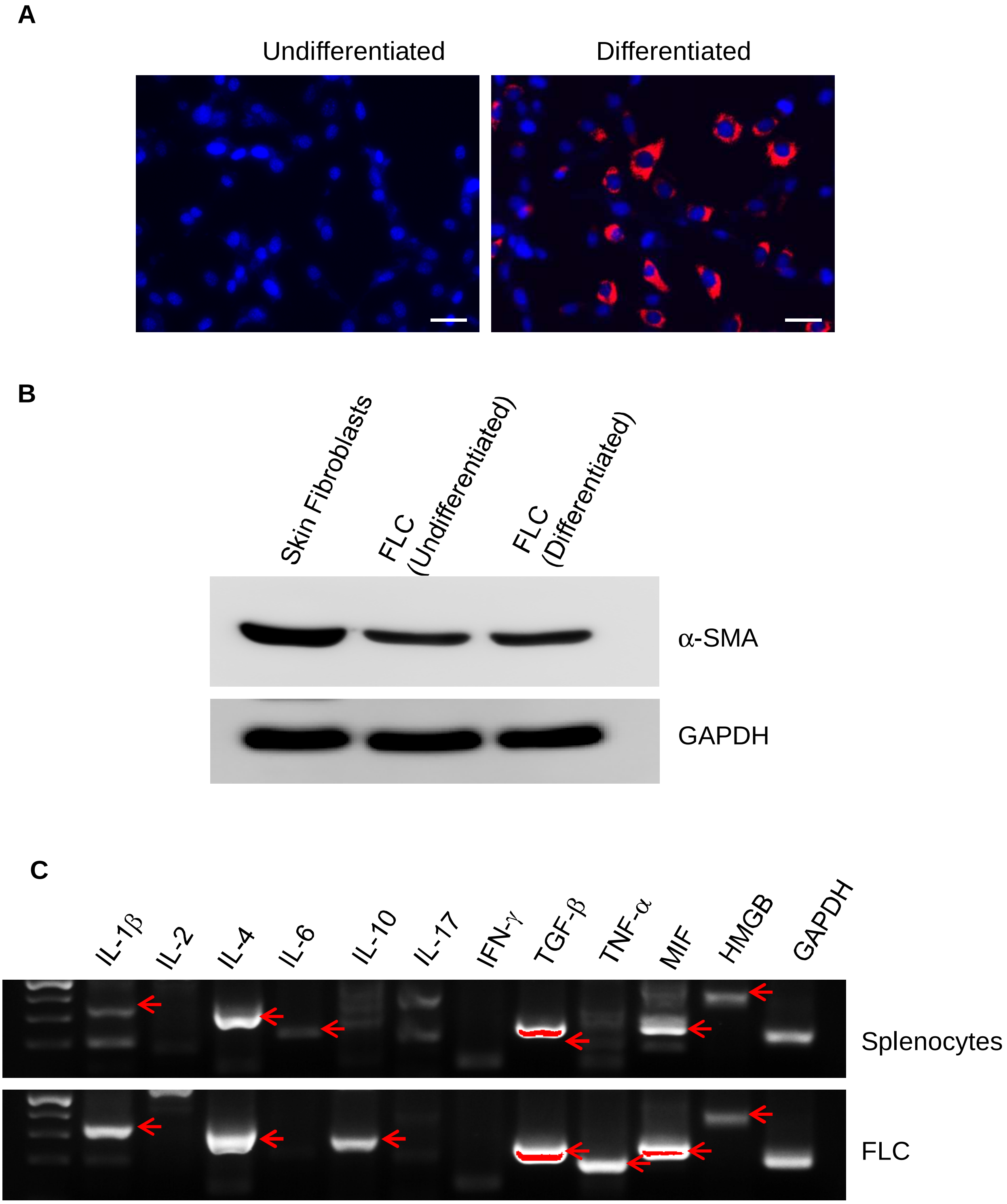
A
Undifferentiated
Differentiated
B
Skin Fibroblasts
FLC (Undifferentiated)
FLC (Differentiated)
α-SMA
GAPDH
C
IL-1β
IL-2
IL-4
IL-6
IL-10
IL-17
IFN-γ
TGF-β
TNF-α
MIF
HMGB
GAPDH
Splenocytes
FLC

Figure S5

Figure S5: The possible mechanisms of SSEA-positive multipotent stem cells contributing to skin wound healing. (**A**) SSEA-positive stem cells can differentiate into type 1 pro-collagen producing fibroblasts. Splenocytes-derived stem cells induced by M-CSF were induced to differentiation by culture cells in fibroblast conditioned medium for two weeks[24] . The undifferentiated and differentiated cells were stained with type 1 pro-collagen antibody (red color). DAPI was used for cellular nucleus staining. Scale Bars, 50 μm. (**B**) The undifferentiated and differentiated mouse splenocyte-derived stem cells (FLC) express α-smooth muscle actin detected by western blotting. Skin fibroblasts were used as a positive control. GAPDH was used for a loading control. (**C**) Splenocyte-derived stem cells express cytokines contributing to the wound healing. RNA was extracted from mouse splenocytes (top panel) or splenocytes-derived stem cells (FLC, bottom panel) and cDNA were synthesized. Reverse transcript PCR was peformed for 35 cycles. Arrows indicated PCR products with the expected size for different cytokine. There were remarkable higher expressions of IL-1β, IL-10 and TNF-α in splenocyte-derived stem cells (FLC) compared to that in splenocytes.
